# Genome-Scale Analysis of the Grapevine KCS Genes Reveals Its Potential Role in Male Sterility

**DOI:** 10.3390/ijms24076510

**Published:** 2023-03-30

**Authors:** Huan Zheng, Yueting Liang, Ben Hong, Yingyi Xu, Mengfan Ren, Yixu Wang, Liyuan Huang, Lina Yang, Jianmin Tao

**Affiliations:** 1 College of Horticulture, Nanjing Agricultural University, Nanjing 210095, China; 2 Charles River Laboratories International, Inc., Michigan, MI 01887, USA

**Keywords:** grapevine, *VvKCS*, genome-wide, male sterility, very long-chain fatty acid

## Abstract

Very long-chain fatty acid (VLCFA) synthesis in plants, is primarily rate-limited by the enzyme 3-ketoacyl CoA synthase (KCS), which also controls the rate and carbon chain length of VLCFA synthesis. Disruption of VLCFA during pollen development, may affect the pollen wall formation and ultimately lead to male sterility. Our study identified 24 grapevine *KCS* (*VvKCS*) genes and provided new names based on their relative chromosome distribution. Based on sequence alignment and phylogenetic investigation, these genes were grouped into seven subgroups, members of the same subgroup having similar motif structures. Synteny analysis of *VvKCS* genes, showed that the segmental duplication events played an important role in expanding this gene family. Expression profiles obtained from the transcriptome data showed different expression patterns of *VvKCS* genes in different tissues. Comparison of transcriptome and RT-qPCR data of the male sterile grape ‘Y−14’ and its fertile parent ‘Shine Muscat’, revealed that 10 *VvKCS* genes were significantly differentially expressed at the meiosis stage, which is a critical period of pollen wall formation. Further, joint analysis by weighted gene co-expression network analysis (WGCNA) and Kyoto Encyclopedia of Genes and Genomes (KEGG), revealed that five of these *VvKCS* (*VvKCS6/15/19/20/24*) genes were involved in the fatty acid elongation pathway, which may ultimately affect the structural integrity of the pollen wall in ‘Y−14’. This systematic analysis provided a foundation for further functional characterization of *VvKCS* genes, with the aim of grapevine precision breeding improvement.

## 1. Introduction

China is the largest producer of table grapes, but the main varieties used in the production, such as ‘Kyoho’, ‘Summer Black’, and ‘Shine Muscat’, are bred in Japan. With increasing demand for variety diversity and the restrictions of new variety rights in foreign countries, the selection and breeding of new high-quality seedless grape varieties have become important breeding objectives [1]. The way to obtain seedless grapes in production, is either by choosing seedless varieties or by using plant growth regulators for seedless treatment. The formation of seedless grapes, whether stimulated by genetic or extrinsic factors, requires parthenocarpy ability, and male sterility is one of the important genetic factors leading to seedless fruit. Using male sterile plants as the maternal parent for hybrid breeding, can eliminate the process of stamen removal, simplify the breeding procedure, and improve efficiency [2]. Therefore, research on the mechanism of male sterility in grapes can provide a theoretical basis for new variety breeding and production practice.

VLCFAs are fatty acids longer than 18 carbons (C18) in length, which are required in all plant cells for the production of sphingolipids [3]. In addition, VLCFAs could be derivatized, modified, or esterified to other molecules, or polymerized to create physiologically active molecules, such as fatty acids, aldehydes, alcohols, ketones, alkanes, wax esters, tryphine precursors, flavonoids, and sporopollenin precursors [4,5]. Previous studies have shown that VLCFAs play key roles in plant reproductive development, especially in pollen wall and anther cuticle development [6,7], as well as other aspects of reproductive development, such as anther dehiscence [8] and pollen maturation [9], pollen hydration [10,11], flowering [12], and pollen tube growth [13]. Any changes in VLCFA metabolism may affect reproductive development or lead to male sterility.

VLCFAs are synthesized by fatty acid elongation (FAE) complexes in the endoplasmic reticulum (ER), which are synthesized through four sequential reactions, catalyzed by 3-ketoacyl-CoA reductase (KCR), 3-ketoacyl-CoA synthase (KCS), enoyl reductase (ECR), and 3-hydroxyacyl-CoA dehydratase (HCD). Among all the reactions, KCS is the only enzyme in the FAE complex that catalyzes a reaction unique to a certain substrate, whereas the other three enzymes are generalists [4]. Thus, KCS functions as a rate-limiting enzyme, and determines the length of the final acyl-CoA products [4]. Because of this, KCSs have expanded into a large, multigenic family, with diverse substrates, yet to be fully explored [14]. Twenty-one *KCS* sequences have been identified in the *Arabidopsis* genome, part of which was reportedly involved in reproductive development [14]. For example, *KCS6* (also known as *CER6*, *POP1*, and *CUT1*) and *KCS10*, were widely expressed in *Arabidopsis*. The *KCS6* and *KCS10* mutants showed lower wax content on pollen surfaces, which affects stigma recognition for pollen, and leads to male sterility [10]. As there is overlap in both the expression patterns and substrate specificities of many *Arabidopsis KCSs*, there is likely functional redundancy within the family [14]. Indeed, this has already been confirmed for several groups of condensing enzymes. For example, KCS2, KCS9, and KCS20 all elongate saturated VLCFAs for cuticular wax and suberin biosynthesis [4].

In this study, 24 *KCS* genes in grapevine were identified by genome-wide analysis technology. The chromosome location, phylogenetic relationships, gene structure, gene synteny, motif compositions, *cis*-elements, and tissue expression patterns were explored from a full perspective. In addition, we used RNA-seq data from male sterile and normal fertility grapes, to screen for differentially expressed genes of *VvKCS*. WGCNA and KEGG analysis were used to identify *VvKCS* genes that may be involved in fatty acid chain elongation reactions during the meiosis stage. This information may be a theoretical basis for elucidating the mechanism responsible for *VvKCS* gene family-mediated reproductive development in the grapevine.

## 2. Results

### 2.1. Identification and Annotation of KCS Gene Family Members in Grapevine

Twenty-four *VvKCS* genes were identified in the grapevine genome, by identifying and validating the structural domains in the genome-wide protein database. A total of 21 *VvKCS* genes could be mapped on the linkage groups. These genes were renamed, from *VvKCS1* to *VvKCS21*, based on their order in the linkage groups. Three *VvKCS* genes that could not be conclusively mapped to any linkage groups were renamed *VvKCS22*–*VvKCS24*, respectively (Table S1). As shown in Table S1, the 24 *VvKCS* family genes were unevenly distributed on the chromosomes. Among them, *VvKCS* genes were not present on chromosomes 2, 17, and 18, while they were most numerous on chromosomes 7 and 16, which both contained three *VvKCS* genes. There were two *VvKCS* genes on chromosome 5, and one on each of the remaining chromosomes. Therefore, the *VvKCS* gene family members are have a relatively scattered distribution about the chromosomes. Gene characteristics, including chromosome location, strands, and gene length, are shown in Table S1, in which the longest gene was *VvKSC2*, with 3538 bp and the shortest was *VvKSC24*, with 744 bp.

### 2.2. Phylogenetic Motif Composition and Structural Analysis of VvKCS Genes

**Figure 1:**
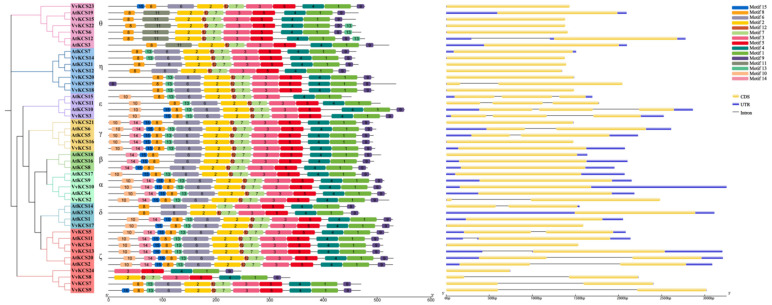
Phylogenetic gene structure and conserved motif analysis of VvKCSs. Based on amino acid sequence alignments of grapevine and *Arabidopsis* VvKCSs, an unrooted neighbor-joining (NJ) tree for VvKCS proteins was created. All the proteins were divided into eight clades, which are colored differently (shown in the left picture). Conserved motifs of grapevine VvKCS proteins are shown in the middle picture. Fourteen conserved motifs are depicted in different colored boxes, as indicated on the right of the figure, exon-intron structures of *VvKCS* genes are shown in the right-hand picture, in which the yellow boxes represent coding sequences (CDS), the blue boxes represent upstream/downstream regions (UTR), and black lines represent introns. The lengths of proteins/genes can be estimated using the scale at the bottom.

To investigate the evolutionary relationships of the *VvKCS* genes, a phylogenetic analysis of the *VvKCS* and *AtKCS* genes was performed. The 21 AtKCS and 24 VvKCS protein sequences, were aligned using Clustal W (version 2.1), and an unrooted phylogenetic tree was constructed using the neighbor-joining method. All KCS proteins were classified into eight subgroups, α to θ (Figure 1). As shown in Figure 1, the β subgroup only consisted of *Arabidopsis* KCS proteins. The α and ε subgroups contained two VvKCS members. The γ subgroup contained three VvKCS members. The δ subgroup contained only one VvKCS member. The ζ subgroup has the most members (seven VvKCS members). The η subgroup contained five VvKCS members. The θ subgroup contained four VvKCS members.

Gene structures have generally diversified during the evolution of numerous gene families. In order to expand our understanding of VvKCSs in the evolution and functional diversification, we analyzed the motifs and gene structure using the MEME analysis tool and GSDS 2.0, respectively. As shown in Figure 1, each VvKCS contained 5 to 14 motifs, and motif 1, motif 3, motif 4, and motif 5 were presented in all *VvKCS* genes. VvKCS24 contained a minimum of five motifs, and VvKCS1 and VvKCS16, in the γ subgroup, contained fourteen motifs. Most of the functions of these conserved motifs remain to be elucidated. Genes clustered into the same subgroups have similar conserved motifs.

The gene structure mainly comprises exons, introns, and non-coding regions (UTR). Gene structure analysis revealed that *VvKCS* genes clustered in subgroup θ, γ, and δ have no introns, *VvKCS* genes clustered in subgroup ζ have one intron, and *VvKCS* genes clustered in subgroup ε have a more complex structure, which contains two introns. In general, genes clustered in the same subgroup have similar conserved motifs and gene structures, which, together with the results of the phylogenetic analysis, could strongly support the credibility of the group classifications.

### 2.3. Synteny Analysis of VvKCS Genes

**Figure 2:**
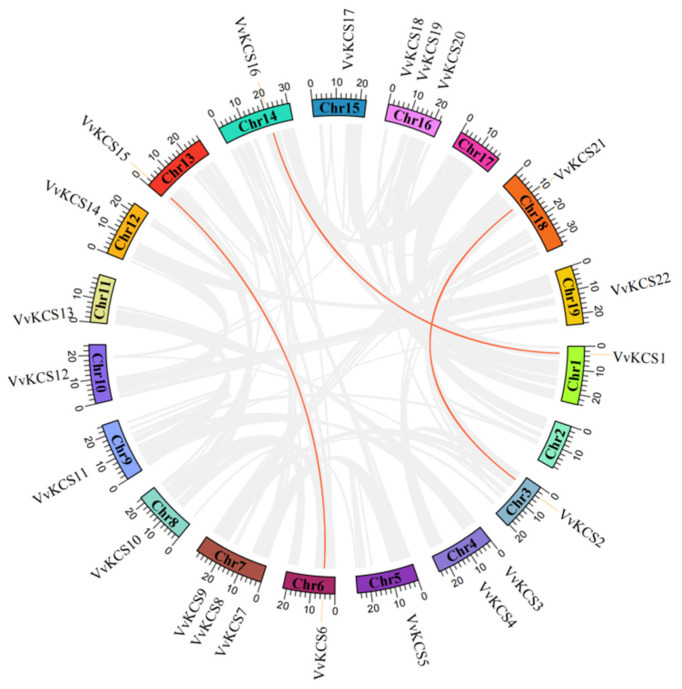
Synteny relationships of *VvKCS* genes in grapevine. The outer circle segment represents the grapevine chromosomes. Link lines in the circle represent paralogous gene pairs between *VvKCSs*.

During evolution, gene families are amplified by tandem and segmental duplication, to enhance plant adaptation to the environment [15,16]. According to previous research, different members of the same gene family occurring within the same or neighboring intergenic regions, could be defined as tandem events [17]. However, multiple genes, through polyploidy, followed by chromosome rearrangements, could be defined as segmental events [18]. We performed a synteny analysis of the grapevine *VvKCS* gene family using the MCScanX software. In this study, three *VvKCS* gene pairs (*VvKCS1/16*, *VvKCS2/21*, *VvKCS6/15*) were identified as segmental duplication, and they were localized on chromosomes 1, 3, 6, 13, 14, and 18 (Figure 2). These results indicated that some *VvKCS* genes were possibly generated by gene duplication, and the segmental duplication events were an important driving force in *VvKCS* gene evolution.

**Figure 3:**
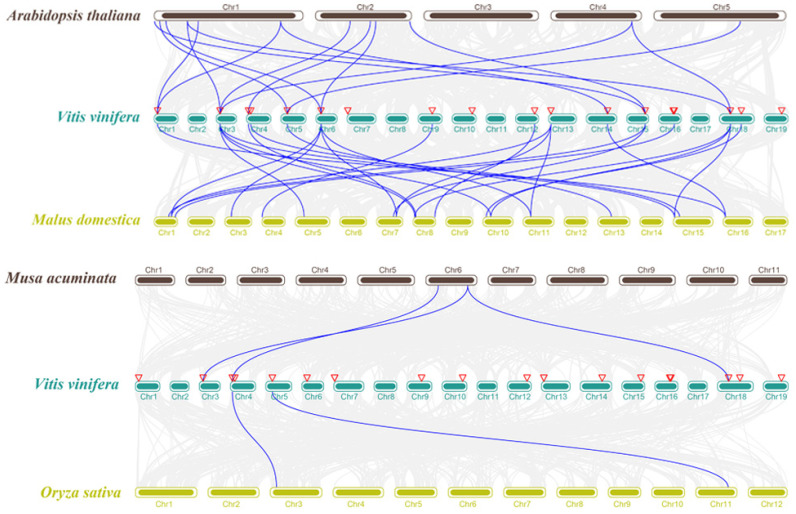
Synteny analysis of *VvKCS* genes between grapevine and four representative plant species. The grey lines in the background represent collinear blocks within the grapevine and other plant genomes, whereas the blue lines indicate syntenic *VvKCS* gene pairs. The red triangle represents the location of *VvKCSs* genes.

Furthermore, two comparative syntenic maps of grapevine, associated with four representative plant species (two dicots: *Arabidopsis*, *Malus domestica*; two monocots: *Oryza sativa*, *Musa acuminata*) were constructed, to illustrate the evolution mechanism of the *VvKCS* gene family. Fourteen *VvKCS* genes showed a syntenic relationship with those in *Arabidopsis*, *Malus domestica*, *Oryza sativa*, *and Musa acuminata*, respectively. Twenty-five and fifteen orthologous pairs were found between *Arabidopsis* and *Malus domestica*, respectively (Figure 3). Three and two orthologous pairs were found between *Musa acuminata* and *Oryza sativa*, respectively. Some *VvKCS* genes were found to be associated with at least three syntenic gene pairs, such as *VvKCS2/3/5/10*. We guessed these genes may have played an important role in the *VvKCS* gene family during evolution. Additionally, *VvKCS3* was identified between grapevine and the other four species, indicating that these orthologous pairs may have already existed before the ancestral divergence (Table S2).

### 2.4. Expression of VvKCS Genes in Different Tissues of Grapevine

**Figure 4:**
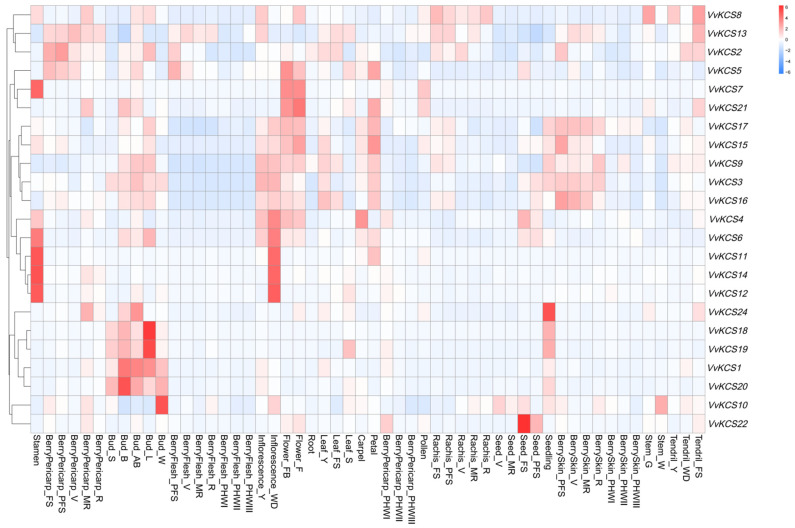
Expression profiles of the *VvKCSs* in 54 tissues of grapevine. Please refer to the website (https://www.ncbi.nlm.nih.gov/geo/geo2r/?acc=GSE36128 accessed on 19 March 2023) for a specific explanation of letter designations.

To comprehensively understand the *VvKCS* genes, we used RNA-seq data from the NCBI (SRA database, NO: GSE36128), to study the tissue-specific expression of grapevine *VvKCS* genes. We found that 23 *VvKCS* were specifically expressed in different tissues, by examining *VvKCS* genes at the transcriptional level in 54 different tissues, and some genes had similar expression patterns. For example, *VvKCS11/14/12* were all highly expressed in stems and flowers but had low expression levels in other tissues, suggesting that these genes may be functionally similar. We have focused on reproduction-related tissues and found that *VvKCS3/4/6/9/15/16/17* were highly expressed in periods of Inflorescence_Y, Inflorescence_WD, Flower_FB, and Flower_F. Except for these, *VvKCS8/10/11/12/13/14* were highly expressed in Inflorescence_Y, or Inflorescence_WD stages, and *VvKCS5/7/8/21/22* were highly expressed in Flower_FB and Flower_F stages. We also found that *VvKCS* genes were generally expressed at low levels in pollen, with slightly higher expression levels of *VvKCS7/8/13/15/21* in comparison. Interestingly, we found that the expression of *VvKCS4/6/7/8/11/12/14/15* was relatively high in stamens (Figure 4). These results suggest that the above *VvKCS* genes could be involved in the regulation of reproductive growth.

### 2.5. Analysis of Cis-Acting Elements in the Promoter Regions of VvKCS Genes

**Figure 5:**
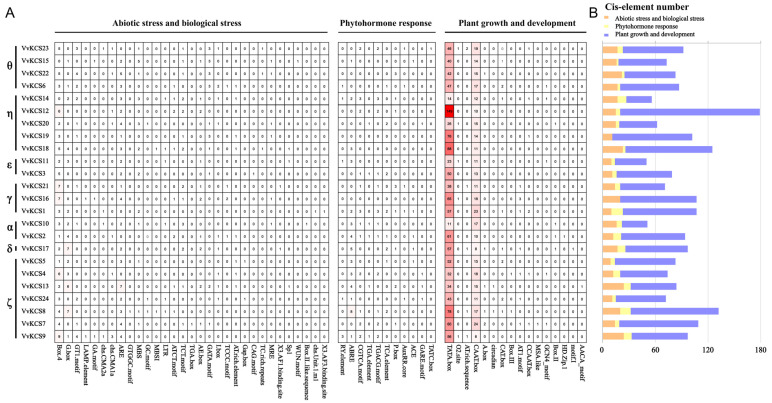
Prediction of *cis*-acting elements in the promoters of grapevine *VvKCSs.* (**A**) The 2000 bp sequence upstream of the *VvKCSs* promoters was analyzed, and the number of *cis*-acting elements is shown in the figure; (**B**) the total number of *cis*-acting elements belongs to abiotic/biotic stress, phytohormone responses, and plant growth and development for *VvKCSs.*

*Cis*-acting element prediction in promoters, will greatly aid knowledge of *VvKCS* functions in the grapevine. Transcription factors (TFs) are an essential class of regulators involved in the transcriptional regulation of gene expression. *Cis*-acting elements in the promoter are critical portions of the transcription factor binding site, for starting transcription and gene expression. To obtain an insight into the putative regulation mechanism of TFs in the expression of *VvKCSs*, PlantCARE was used, to predict the *cis*-acting regulatory elements in the 2000 bp upstream promoter regions of all *VvKCSs*. All of the *cis*-acting elements in *VvKCSs* promoters were categorized into three groups: abiotic/biotic stress, phytohormone, and growth and development (Figure 5A). The average number of *cis*-acting elements associated with growth and development (69) was the largest, followed by abiotic/biotic stress (16) and phytohormones (6) (Figure 5B). As shown in Figure 5A, the *cis*-acting elements TATA box and CAAT box were present in all *VvKCS* gene family members. Previous studies have revealed that endogenous hormones play a pivotal role in plant stamen fertility. In this study, we also found many related *cis*-elements in the promoters of *VvKCS* genes, such as gibberellin-responsive element GARE-motif, auxin-responsive element TGA-element, salicylic acid responsiveness TCA-element, and MeJA-responsiveness TGACG-motif/CGTCA-motif.

### 2.6. Expression Analysis of VvKCS Genes during the Critical Period of Pollen Abortion in Grapevine ‘Y−14’

**Figure 6:**
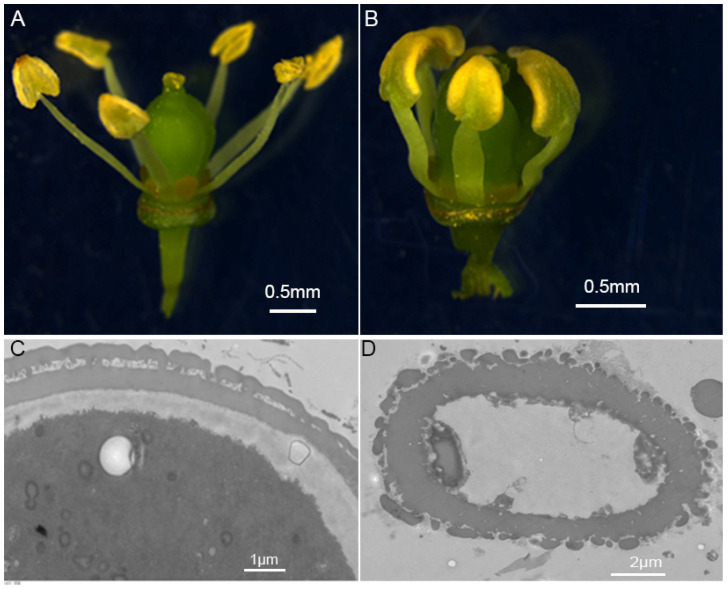
Morphology observation of male sterile grapevine flowers and pollen grains. (**A**) The phenotype of ‘Shine Muscat’; and (**B**) ‘Y−14’ flowers. Transmission electron microscopic observation of (**C**) ‘Shine Muscat’; and (**D**) ‘Y−14’ pollen grains.

‘Y−14’ is a new grapevine germplasm, selected from the self-progeny of the cultivar ‘Shine Muscat’ [19]. This new germplasm exhibited an abnormal phenotype, with ‘Y−14’ filaments being extremely short, stamens tightly wrapped around the pistil, and anthers that do not undergo dehiscence easily (Figure 6A,B). The pollen viability assay showed that ‘Y−14’ pollen grains did not germinate, while the germination rate of ‘Shine Muscat’ pollen grains reached 72.3% (Table 1). Using transmission electron microscopy, we found that the cellular contents of ‘Y−14’ disappeared. The tryphine and the tectum layers on the external wall of pollen grains were incomplete, in contrast to ‘Shine Muscat’, where the pollen grains were enriched with dense contents. The external wall of pollen was structurally intact (Figure 6C,D). Bagged before flowering, the ‘Y−14’ produced seedless berries, and cross-pollination with fertility pollen could yield normal seeds. The above results indicated that ‘Y−14’ is a classic male sterile germplasm.

**Figure 7:**
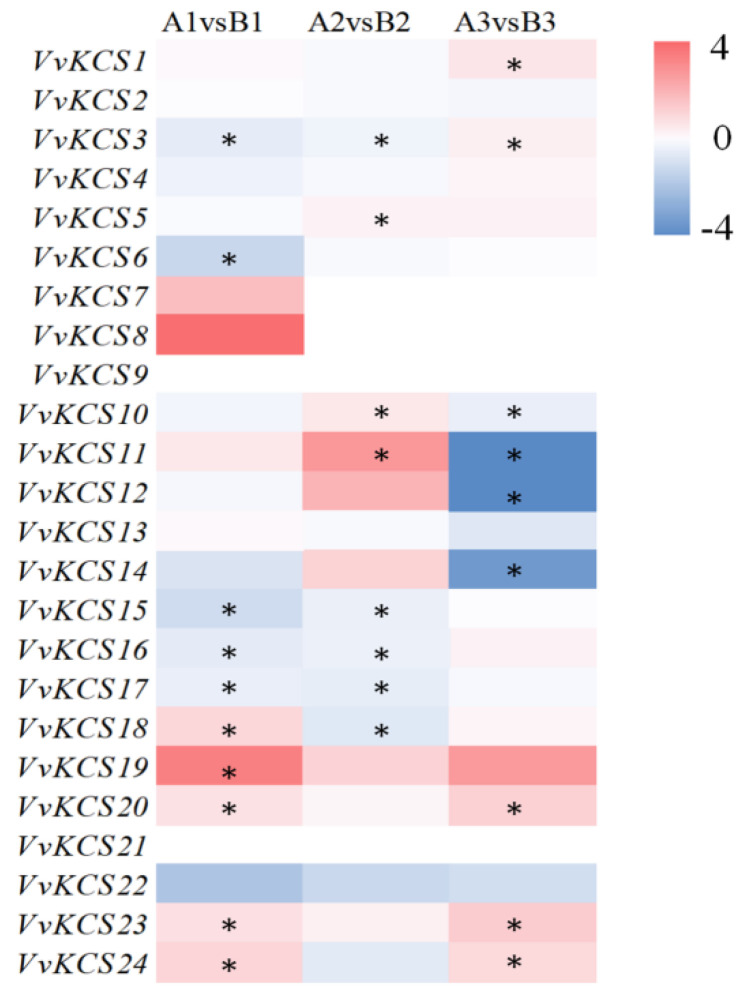
The heatmap for each class depicts the changes in the expression of various *VvKCS* genes. The color legend depicts the log_2_-fold variation in the FPKM ‘Y−14’/FPKM ‘Shine Muscat’ ratio. A positive value indicates that the gene was upregulated in the Y−14, whereas a negative value indicates that the gene was downregulated in the Y−14. Student’s t-test was used to determine statistical significance. *, *p* ≤ 0.05. A: ‘Y−14’; B: ‘Shine Muscat’. 1: meiosis stage; 2: tetrad stage; 3: early mononuclear stage.

Previous studies found that pollen wall formation starts at the meiosis stage, when the callose surrounding microspores are degraded by cellase secreted from the tapetum [5]. To further explore the potential roles of *VvKCS* genes in pollen development, the expression of all the *VvKCS* genes was examined in the transcriptome sequencing data of pollen, from the meiosis, tetrad, and mononuclear early stages. Among the 24 *VvKCS* genes, *VvKCS21* was not expressed in any of the detected samples; this may be because it had special temporal and spatial expression patterns, not examined in our libraries. Seventeen *VvKCS* genes were expressed in all 18 samples tested (FPKM > 0), and 16 genes showed constitutive expression (FPKM > 1 in all samples). We drew violin plots, to see the overall expression based on the FPKM values of the *VvKCS* genes. As shown in Figure S1, the average expression level of *VvKCS* was higher in ‘Y−14’ than in ‘Shine Muscat’ at the meiosis stage, and the FPKM data were less discrete. At the tetrad stage, the mean values and distribution of FPKM values were similar between ‘Y−14’ and ‘Shine Muscat’. In the early mononuclear stage, the mean and discrete degree of ‘Y−14’ was slightly higher than that of ‘Shine Muscat’. Heatmaps of *VvKCS* genes were generated for the meiosis, tetrad, and early mononuclear stages, using the expressed genes (Figure S2). The expression of some genes exhibited significant trends in different development stages. For example, the expression levels of *VvKCS2/13/15/16/17* were gradually increased along with the pollen development. However, some *VvKCS* genes showed opposite trends in fertile and sterile materials. For example, *VvKCS3* expression increased with pollen development in ‘Y−14’ but decreased in ‘Shine Muscat’ (Figure S2). The log_2_ (FPKM_‘Y−14’/FPKM_’Shine Muscat’) values were used to make a heatmap, to better compare the expression of *VvKCS* genes in fertile and sterile samples. As shown in Figure 7, at meiosis, *VvKCS3/6/15/16/17* were significantly decreased, and *VvKCS18/19/20/23/24* were significantly increased in ‘Y−14’, as compared to ‘Shine Muscat’. At the tetrad stage, ‘Y−14’ showed significantly higher expression of *VvKCS5/10/11*, but significantly lower expression of *VvKCS3/15/16/17/18*, than ‘Shine Muscat’. Significantly higher *VvKCS1/3/20/23/24* genes and significantly lower *VvKCS10/11/12/14* were observed in ‘Y−14’ samples, as compared to ‘Shine Muscat’ at the early mononuclear stage (Figure 7). The reliability of the transcriptome data was further validated by quantitative real-time PCR (RT-qPCR) experiments for eight selected *VvKCS* genes (Figure S3).

### 2.7. Screening for Key VvKCS Genes Regulating Pollen Development by WGCNA

**Figure 8:**
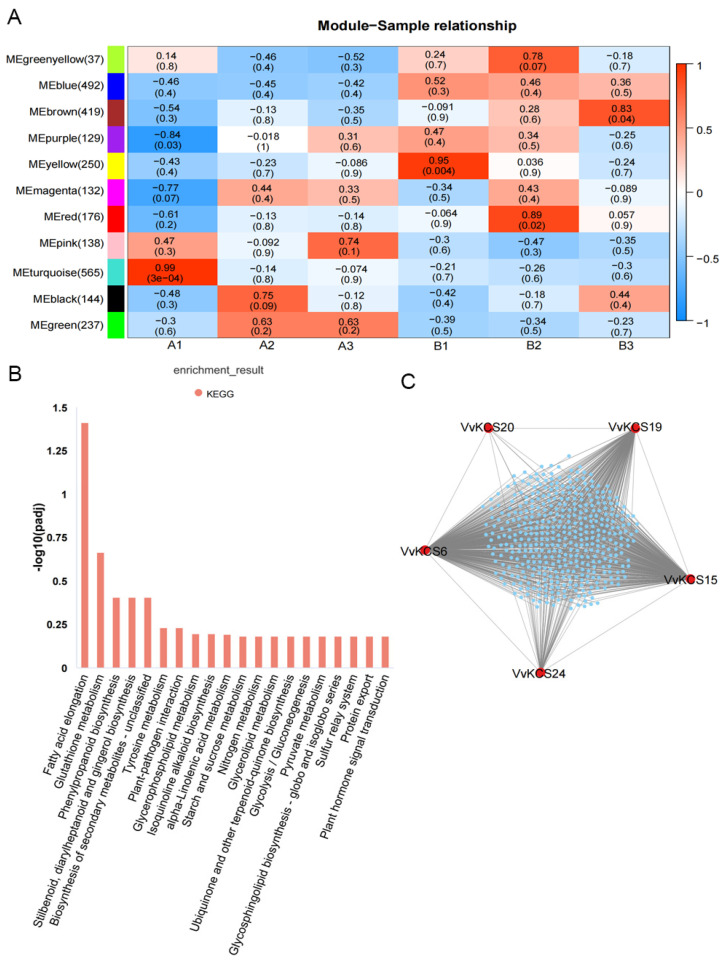
Screening of key *VvKCS* genes regulating fatty acid chain elongation by WGCNA and KEGG analyses. (**A**) Module–sample relationship and corresponding *p*-values. The left panel shows the 11 modules and the numbers in parentheses represent the total number of genes within each module. The color scale on the right illustrates the module trait correlation, ranging from −1 (blue) to 1 (red). A1: ‘Y−14’ of meiosis stage. A2: ‘Y−14’ of the tetrad stage. A3: ‘Y−14’ of the early mononuclear stage. B1: ‘Shine Muscat’ of the meiosis stage. B2: ‘Shine Muscat’ of the tetrad stage. B3: ‘Shine Muscat’ of the early mononuclear stage; (**B**) ‘Y−14’ of the meiosis stage was significantly and positively correlated with the turquoise module; KEGG analyzed the genes inside the module; (**C**) computed regulatory network of the five *VvKCS* genes in the fatty acid elongation pathway. Blue dots represent genes that may have an interactive relationship with *VvKCS* genes.

Gene co-expression networks were constructed, using transcriptome data from three key pollen developmental periods of ‘Y−14’ and ‘Shine Muscat’. Eleven co-expression modules were obtained for ‘Y−14’ and ‘Shine Muscat’ materials, by merging similarly expressed modules, using the dynamic cut-tree method on weight values. Different colors represent different modules (Figure S4). The gene expression levels in the 11 co-expressed gene modules and the three-time points of pollen development, were correlated for analysis. As shown in Figure 8A, the horizontal axis is the developmental period of the sample, and the vertical axis is the gene module. Each unit consists of the corresponding correlation value and *p* value; positive correlation is marked in red, while negative correlation is marked in blue. Among them, the turquoise (r = 0.99, *p* = 3 × 10^−4^) and purple (r = −0.84, *p* = 0.03) modules showed a strong correlation with the samples at the meiosis stage of ‘Y−14’. The expression levels of genes in the yellow (r = 0.95, *p* = 0.004), red (r = 0.89, *p* = 0.02), and brown (r = 0.83, *p* = 0.04) modules were significantly and positively correlated with the meiotic stage, tetrad stage, and early mononuclear stage of the ‘Shine Muscat’ samples, respectively (Figure 8A). KEGG enrichment pathway analysis of these significantly related modules, revealed that the turquoise module was most significantly enriched in the fatty acid elongation (Figure 8B and Table S3), while the yellow module was mainly enriched in the glycerolipid metabolism, lysine degradation, tryptophan metabolism, cutin, suberine, and wax biosynthesis pathways, and so on. The red module was mainly enriched in the phosphatidylinositol signaling system pathway. The brown module was mainly enriched in the cysteine and methionine metabolism and nitrogen metabolism pathways (Table S3). The pollen wall of ‘Y−14’ is incomplete (Figure 6), and significantly and positively correlated with the turquoise module during the meiosis stage (Figure 8A), which is the key period of pollen wall formation. We focused on the fatty acid elongation pathway in the turquoise module and found that all five enriched genes belonged to the *VvKCS* gene family (*VvKCS6/15/19/20/24*) (Table S3). Cytoscape was used to map the gene interaction network of *VvKCS* genes in the turquoise module, and it was found that *VvKCS6* was at the hub position, with the highest connectivity, followed by *VvKCS15* and *VvKCS19*, and *VvKCS20* had the lowest connectivity (Figure 8C). All these results suggest that some members of the *VvKCS* gene family were involved in the regulation of ‘Y−14’ pollen development. Abnormal expression at the meiosis stage may affect the fatty acid elongation in pollen grains, leading to pollen grain abortions.

## 3. Discussion

### 3.1. Systematic Analysis Lays the Foundation for Functional Characterization of the VvKCS Gene

The *KCS* gene family, which is extensively distributed in plants, encodes enzymes that catalyze the rate-limiting condensation step, and hence particularly govern the elongation of fatty acids. Therefore, they will probably be very important for substrate and product specificity. The *KCS* gene family has been discovered and examined at the genome-wide level of numerous plant species, due to its significant function in VLCFA synthesis [20]. Here, 24 *VvKCS* in grapevine were identified, using comparative genomics approaches (Table S1). Phylogenetic, gene structure, and conserved domain/motif analyses showed similar patterns and the same conserved domain in all *VvKCSs* (Figure 1).

In this study, three *VvKCS* gene pairs (*VvKCS1/16*, *VvKCS2/21*, *VvKCS6/15*) were identified as duplication, and localized on chromosomes 1, 3, 6, 13, 14, and 18 (Figure 2). Since these genes were not in the same or adjacent intergenic regions; thus, we inferred that some *VvKCS* genes were possibly generated by gene duplication, and the segmental duplication events were an important driving force in *VvKCS* gene evolution. Evolution events, including segmental duplication, have extended the gene families’ members in plant species, and mutations in the coding sequences and promoter regions might affect the expression and function of new members [21,22].

We further analyzed the structures and expression patterns of these three paralogous gene pairs. Then, we found that the sequence length of the UTR region of *VvKCS1* was significantly longer than that of *VvKCS16* (Figure 1). *VvKCS1* was mainly expressed in the bud, but *VvKCS16* was also highly expressed in other tissues, such as berry skin and the bud (Figure 4). *VvKCS6* and *VvKCS15* are distinguished by one motif, and these two genes have similar expression patterns and are mainly highly expressed in reproductive tissues. *VvKCS2* has one more intron than *VvKCS21*, but the UTR of *VvKCS21* was longer than that of *VvKCS2*, and the expression patterns of the two genes were significantly different, with *VvKCS2* expressed at high levels in more tissues than *VvKCS21*. Previous research on non-coding areas has discovered a strong negative association between intron length and the level of related gene expression. The intron length of genes with low levels of expression was noticeably longer than that of genes with high levels of expression. An increase in the number of introns will increase the structural number of nuclear localization signal protein motifs, affecting the levels of protein entering the nucleus and the effectiveness of DNA binding [23]. Therefore, these results suggest that, although they are paralogous genes, structural variants, especially in non-coding regions, lead to changes in expression patterns, which may lead to functional differentiation.

Analysis of synteny between grapevine and other species, showed that *VvKCS2/3/5/10* exist in monocotyledonous and dicotyledonous plants as related orthologous gene pairs (Figure 3 and Table S2). These results suggest that these four genes were present before mono/dicotyledonous differentiation, and that they may exercise relatively important and functionally conserved functions. Analysis of synteny between grapevine and dicots, revealed that the number of orthologous gene pairs was greater in grapevine and *Malus domestica*, than in grapevine and *Arabidopsis*, the result that also supports a later differentiation process in grapevine and *Malus domestica* than in *Arabidopsis* [24]. Among them, *VvKCS1/2/3/5/6/10/16/17* were present in orthologous gene pairs in both *Malus domestica* and *Arabidopsis*, indicating that these genes are relatively conserved in dicotyledons and may exercise similar functions. *KCS* gene function was relatively clear in the model plant *Arabidopsis*, but much less information was available in grapevine. Therefore, these syntenic relationships could inform our subsequent in-depth studies of the *VvKCS* genes. It has been reported that *KCS6* (also known as *CUT1*, *CER6*, or *POP1*) was mainly expressed in leaves, stems, and flowers, and was required for the production of VLCFAs [10]. The *cer6* mutant displayed the absence of all classes of long-chain lipids in the stem cuticle and pollen coat, thus having no pollen coat. Pollen grains of the mutants were defective in hydration and displayed male sterility in a low-humidity environment, but the male sterility could be rescued in high-humidity conditions.

Furthermore, research found that *AtKCS6* catalyzes the production of C28 fatty acyl-CoAs [4]. *HMS1*, the *Arabidopsis KCS6* homologue, is critical for catalyzing the biosynthesis of the C26 and C28 VLCFAs, contributing to the formation of bacula and tryphine in the pollen wall, which protects the pollen from dehydration. Under low-humidity conditions, *hms1* pollen showed poor adhesion and reduced germination on the stigmas, which could be rescued by increasing the humidity [25]. The orthologous genes of *AtKCS6* in grapevine were *VvKCS1* and *VvKCS16* (Figure 3 and Table S2). We found that *VvKCS16* was consistently highly expressed at all four stages of floral development (Figure 4) and that *VvKCS16* was significantly lower in both the meiosis and tetrad stages of ‘Y−14’, compared to in ‘Shine Muscat’ (Figure 7). These results suggest that *VvKCS16*, a homolog of *Arabidopsis*, might be involved in pollen abortion in ‘Y−14’.

In addition, we found that *VvKCS3/4/6/9/15/16/17* were consistently highly expressed from the young inflorescence stage to the blooming flower stages, by analyzing the expression of *VvKCS* genes in 54 tissues (Figure 4). *VvKCS5/7/8/11/12/13/14/20/21/22/24* were specifically expressed at a certain stage of flowering or in a specific tissue (pollen or stamen) (Figure 4), suggesting that these genes might also be involved in reproductive development. Phylogenetics, gene structure, conserved domain/motif, *cis*-elements, and expression analyses provided substantial information on the functions and roles of these genes.

### 3.2. VvKCS Genes May Be Involved in Pollen Cell Wall Formation

The pollen wall is important for fertilization, pollen–stigma interaction, and seed development. It also protects male gametophytes from numerous environmental stresses [26]. The pollen wall typically has three layers: the tryphine (pollen coat), the outer exine, and the inner intine [7]. Tryphine, which is mostly made up of complex lipids, flavonoids, waxy esters, pigments, proteins, aromatic chemicals, and other unidentified components, can prevent male gametophytes from drying out and promote pollen–stigma attachment and subsequent communication [7,27]. The exine is made up of the inner nexine and the outer sexine. The nexine has a bilayer structure, consisting of a foot layer and endexine, and the sexine is composed of tectum and bacula. The exine layer consists of the extremely stable sporopollenin, which contains aliphatic monomers, including VLCFAs [6]. In this study, we found that the tryphine and tectum layers on the external wall of ‘Y−14’ pollen grains were incomplete, while the external wall of ‘Shine Muscat’ pollen was structurally intact (Figure 6). Therefore, we assumed that the incomplete structure of the outer wall of pollen was one of the pivotal reasons for ‘Y−14’ abortion.

Previous studies have demonstrated that pollen wall formation starts at the meiosis stage [6]. The callose surrounding the microspores was secreted by cellase, produced from the tapetum. Callose degeneration and young microspores form primexine, a complex template for the assembly and deposition of tryphine and sporopollenin precursors [6,7]. When the endoplasmic reticulum (ER) of the tapetum has finished producing sporopollenin and tryphine precursors, the resulting lipids are swiftly transported onto the pollen surface, via transporters for pollen wall construction [28,29]. VLCFAs and their derivatives are the main components of tryphine and sporopollenin in the pollen wall [5]. As a key rate-limiting enzyme for the synthesis of VLCFAs, we analyzed the expression of *VvKCS* genes in the male sterile material ‘Y−14’ and its fertile parent ‘Shine Muscat’, at the meiosis stage, using RNA-seq data, and found that 10 genes in the *VvKCS* family were significantly differentially expressed in ‘Y−14’, especially *VvKCS3/6/15/16/17* was significantly decreased at the meiosis stage, while *VvKCS18/19/20/23/24* was significantly increased (Figure 7). As for why some of the *VvKCS* genes were elevated and some were decreased in the ‘Y−14’, we hypothesized that these genes might play a role in determining not only the location of VLCFA synthesis but also their length and quantity. Increasing evidence indicates that VLCFA synthesis may determine male fertility, by affecting the formation of pollen, anther, or seeds. For example, *AtKCS18/FAE1* is the first identified *KCS* gene in *Arabidopsis* which is exclusively expressed in seeds, and is involved in the production of lipids used for seed preservation [30,31]. *AtKCS2/DAISY*, *AtKCS9*, *AtKCS10/FDH1*, and *AtKCS20* have been demonstrated to be involved in synthesizing VLCFAs for cuticular waxes [32]. *AtKCS7*, *AtKCS15*, and *AtKCS21* have been shown to be expressed in the tapetum, and played redundant functions in synthesizing the lipids that make up the pollen coat [33]. We performed further analyses, by WGCNA combined with KEGG, and found that several lipid metabolism-related pathways were significantly enriched in the meiosis stage (Figure 8A and Table S3), especially in ‘Y−14’, which was most significantly enriched in the fatty acid elongation pathway (Figure 8A,B). Analysis of genes on this pathway revealed that all five enriched genes belonged to the *VvKCS* gene family (*VvKCS6/15/19/20/24*) (Table S3). Due to the lack of *VvKCSs*-related mutants, we could only identify *VvKCS* genes related to pollen fertility. We will subsequently focus on how these five genes regulate the synthesis of tryphine and sporopollenin in pollen walls.

## 4. Materials and Methods

### 4.1. Genome-Wide Identification of VvKCS Family Genes in Grapevine

Twenty-one KCS proteins in *Arabidopsis* were used to search for the annotated protein sequences in the grapevine genome. The BLASTP search was performed to identify the candidate VvKCS proteins in the grapevine, with a cutoff e-value of 1 × 10^−5^. The duplicate sequences were removed and submitted to InterPro (https://www.ebi.ac.uk/interpro/search/sequence/ accessed on 19 March 2023) online [34], and the presence of the 3-oxoacyl-[acyl-carrier-protein (ACP)] synthase III and Type III polyketide synthase-like domains [14] in each sequence was verified.

### 4.2. Phylogenetic, Motif and Gene Structure Analysis

The KCS amino acid sequences identified from grapevine and *Arabidopsis*, were subjected to multiple sequence alignment using the Clustal W program, in the MEGA-X software. A phylogenetic tree was constructed, using the neighbor-joining method [35]. The robustness of the tree was assessed by the bootstrap method, on 1000 replicates. The conserved amino acid motifs of KCS genes were analyzed by the online software MeMe (http://meme.nbcr.net/meme/intro.html accessed on 19 March 2023). The predicted number of parameters was set at 10, and other parameters were set as default [36]. Gene Structure Display Server 2.0 (gao-lab.org) was used to draw the structure of the KCS gene family [37].

### 4.3. Cis-Acting Elements Analysis of VvKCSs

The DNA sequence of 2000 bp upstream of the *KCS* genes was extracted using the Perl language, and analyzed for *cis*-elements using the Plant CARE software [38]. The obtained results were screened, classified, and graphed using the R language [39].

### 4.4. Gene Duplication and Syntenic Analysis of VvKCSs

All *VvKCS* genes were mapped to grapevine chromosomes based on physical location information from the NCBI database (https://www.ncbi.nlm.nih.gov accessed on 19 March 2023), using Circos [40]. Gene duplication events were analyzed by the Multiple Collinearity Scan toolkit (MCScanX), with the default parameters [41]. Syntenic analysis maps were constructed using the Dual Systeny Plotter software (https://github.com/CJ-Chen/TBtools accessed on 19 March 2023) [42], to exhibit the synteny relationship of the orthologous *VvKCS* genes obtained from grapevine and other selected species.

### 4.5. Expression Analysis of VvKCSs in Grapevine Tissues

Transcriptome data of 54 different tissues of grapevine were obtained from the NCBI (accession number GSE36128) [43]. The expression heatmap of *VvKCS* genes was created using the TBtools software, where the expression levels of genes were normalized using [Log_2_(FPKM+1)] [44]. To further study the potential roles of *VvKCSs* involved in anther and pollen development, the transcriptome sequencing data of pollen at meiosis, tetrad, and early mononuclear stages, were used to examine the expression of all the putative *VvKCSs* in this study. Excel was used to carry out pairwise comparisons of the expression values between the ‘Y−14’ and ‘Shine Muscat’ at each pollen developmental stage, and the findings were subjected to log_2_ transformation.

For real-time RT-qPCR, RNA was obtained and treated with RNase-free DNase (Vazyme, Nanjing, China), to eliminate genomic DNA, then synthesized cDNA with the cDNA Synthesis Kit (Thermo Fisher Scientific, Waltham, MA, USA). Furthermore, we carried out qRT–PCR using SYBR Premix ExTaq (Takara Biotech, Kusatsu, Japan) on a CFX96 Touch™ Real-Time PCR Detection System (Bio-Rad, Hercules, CA, USA). The qPCR regime consisted of an initial denaturation at 94 °C for 4 min, followed by 40 cycles of 95 °C for 10 s, 60 °C for 20 s, and 72 °C for 20 s. Melting curve analysis was performed over the range of 60–95 °C at 0.5 °C intervals. The *VvActin* gene was utilized to normalize the targeted gene. The 2^−ΔΔCt^ technique was then used to calculate the levels of gene expression. As shown in the corresponding figures, all qPCR assays were run with three technical replicates for each biological replicate. Statistical analysis was performed using SPSS (version 21.0), Student’s t-test was used to calculate *p* ≤ 0.05 level of significance. The gene ID and primer sequences used for RT-qPCR are shown in Table S4.

### 4.6. Plant Materials and Phenotypic Analysis of ‘Y−14’

‘Shine Muscat’ and ‘Y−14’ were taken from Tang Shan Cui Gu vineyard, Nanjing Agricultural University, as experimental materials. The vineyards were managed conventionally, and grapevine with consistent tree growth was selected for pollen development studies. Young flower buds were collected from mid-April 2020, once per 3 days, until blooming in early May. Samples at the meiosis, tetrad, and early mononuclear stages of pollen were determined by microscope examination and sent to Novogene for RNA-seq. Flowers were collected at the bloom stage and photographed using a stereo microscope (Leica M165FC, Wetzlar, Germany), to observe the morphological appearance. Pollen viability was determined by the in vitro germination method [2]. For transmission electron microscopy analysis, the pollen grains were fixed in paraformaldehyde containing glutaraldehyde for 12 h, osmium tetroxide for 2 h, dehydrated in acetone in a graded order, embedded in epoxy resin, sectioned at 70 nm by an ultra-thin sectioning machine (Leica), stained with double stains (uranyl acetate, lead citrate), and observed under a transmission electron microscope (Hitachi HT7800, Tokyo, Japan) [26].

### 4.7. RNA Isolation and RNA-seq Data Analysis

Anthers of the ‘Shine Muscat’ and ‘Y−14’ were sampled at the meiosis, tetrad, and early mononuclear stages, with three biological replicates. Total RNA was extracted using TRIzol reagent (TIANGEN, Beijing, China), following the instructions. The Stranded mRNA-seq kit (Vazyme, Nanjing, China) was utilized to create RNA-seq libraries. Next, the Illumina HiSeq platform (HiSeq-PE150) was utilized for sequence analyses in Novogene (Tianjin, China). The raw reads were screened using a Trimmomatic (version 0.33), to eliminate the adaptor and low-quality sequences [45]. STAR (version 2.5.2b) was used to map the clean reads to the grapevine genome [46]. DESeq (Padj.05, version 1.10.1) was used to analyze the transcript assembly and gene expression levels [47]. The WGCNA (version 1.29) package in R, and Cytoscape (version 3.9.1), were used to generate co-expression networks [48]. Clustering the genes identified from WGCNA was performed using KEGG (KOBAS, version 2.0) pathway analyses [49].

## 5. Conclusions

In this study, 24 *VvKCS* genes were identified in the grapevine genome. The chromosomal location, gene structure, phylogenetic classification, synteny analysis, and *cis*-acting elements in the promoter regions of the *VvKCS* genes were investigated. The expression assay of the *VvKCS* in 54 grapevine tissues was analyzed, and screened specifically expressed genes in reproductive tissues. We further found 10 *VvKCS* genes significantly differentially expressed, by comparing the transcriptome data of the male sterile ‘Y−14’ and the fertile parent ‘Shine Muscat’ during the critical period of pollen wall development. Subsequently, we performed further investigations, by WGCNA and KEGG, and found that five of the *VvKCS* genes are involved in the fatty acid elongation pathway at the meiosis stage of ‘Y−14’. Therefore, we hypothesized that these five genes might be involved in the pollen development of ‘Y−14’, and the abnormal expression of these five genes may be an important reason for the incomplete pollen wall of ‘Y−14’. The results given here will be very helpful in further characterizing the biological functions of *KCS* in male sterility.

## Figures and Tables

**Table 1:** Pollen grain viability in ‘Y−14’ and ‘Shine Muscat’.

Name	Total Number of Pollen Grains Tested	Total Number of Germinated Pollen Grains	Germination Rate (%)
‘Y−14’	319	0	0
‘Shine Muscat’	351	254	72.3

## Data Availability

The RNA sequencing datasets generated in this study have been deposited in the National Genomics Data Center with the accession number PRJCA014769 (https://ngdc.cncb.ac.cn/ accessed on 19 March 2023). Other data supporting our findings are available in the manuscript file or from the corresponding author upon request.

## Supplementary Materials

The following supporting information can be downloaded at: https://www.mdpi.com/article/10.3390/ijms24076510/s1.
