# Supplementary material for: Genome-Scale Analysis of the Grapevine KCS Genes Reveals Its Potential Role in Male Sterility
Source: Int J Mol Sci. 2023 Mar 30;24(7):6510. doi: 10.3390/ijms24076510 (PMC10095565; doi:10.3390/ijms24076510)
Supplement: Supplementary file 1 [file ijms-24-06510-s001.zip › SUP.table S4.pdf]

Table S4. Sequences of primers used in this research.

Genes	Primer name	Sequence (5'-3')	GenBank accession No.
VvKCS6	VvKCS6F	AGCCTCGAGCTGACTGAGTA	LOC100241584
	VvKCS6R	CTGTTGCACTTGAACCCTGC	
VvKCS11	VvKCS11F	AGCAGCGAGTGCTCAAGAAT	LOC100256735
	VvKCS11R	TGGTTCACCAACATGGCAGA	
VvKCS14	VvKCS14F	TGGAAGCCGTTACTCCCAAC	LOC100244668
	VvKCS14R	GTGAGACCCCAACAAACCCA	
VvKCS15	VvKCS15F	ACAGCCCGAAAATCGTCCTT	LOC100243402
	VvKCS15R	GGGCGATATCAATGGCGAGA	
VvKCS18	VvKCS18F	GGTGTGGCAGCTTGGAATTG	LOC100244664
	VvKCS18R	ACTTGCGTTTGACAGGCTCA	
VvKCS19	VvKCS19F	ACCTTGCGAAGGTTTGGGAA	LOC100266985
	VvKCS19R	ATATAACCCCCACCACCTCCA	

VvKCS20	VvKCS20F	AGGCCAAAGTTGCTCCTCAA	LOC100258334
	VvKCS20R	ATGTCGCGGCTGAGTGTAAA	
VvKCS24	VvKCS24F	CCCCTAATCCTTCCCGCATC	LOC100251061
	VvKCS24R	TGGCCTCAGCATAGGAGAGT	
VvActin	F	ATGGCAGACGGAGAGGATATTCA	LOC100232866
	R	GCCTTTGCAATCCACATCTGCTG	
