# Supplementary material for: Genome-Scale Analysis of the Grapevine KCS Genes Reveals Its Potential Role in Male Sterility
Source: Int J Mol Sci. 2023 Mar 30;24(7):6510. doi: 10.3390/ijms24076510 (PMC10095565; doi:10.3390/ijms24076510)
Supplement: Supplementary file 1 [file ijms-24-06510-s001.zip › Supplementary figures.pdf]

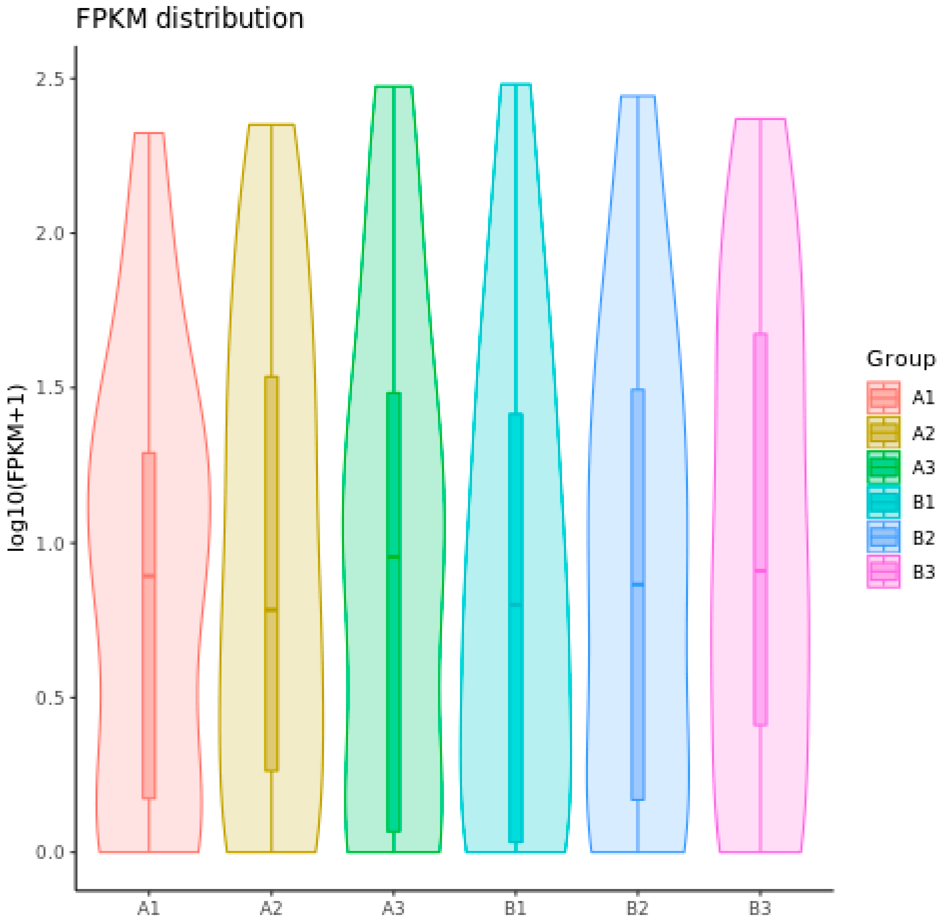

Figure S1. The FPKM distribution of *VvKCS* genes in grapevine. A1, 'Y-14' of meiosis stage. A2, 'Y-14' of the tetrad stage. A3, 'Y-14' of the early mononuclear stage. B1, 'Shine Muscat' of the meiosis stage. B2, 'Shine Muscat' of the tetrad stage. B3, 'Shine Muscat' of the early mononuclear stage.

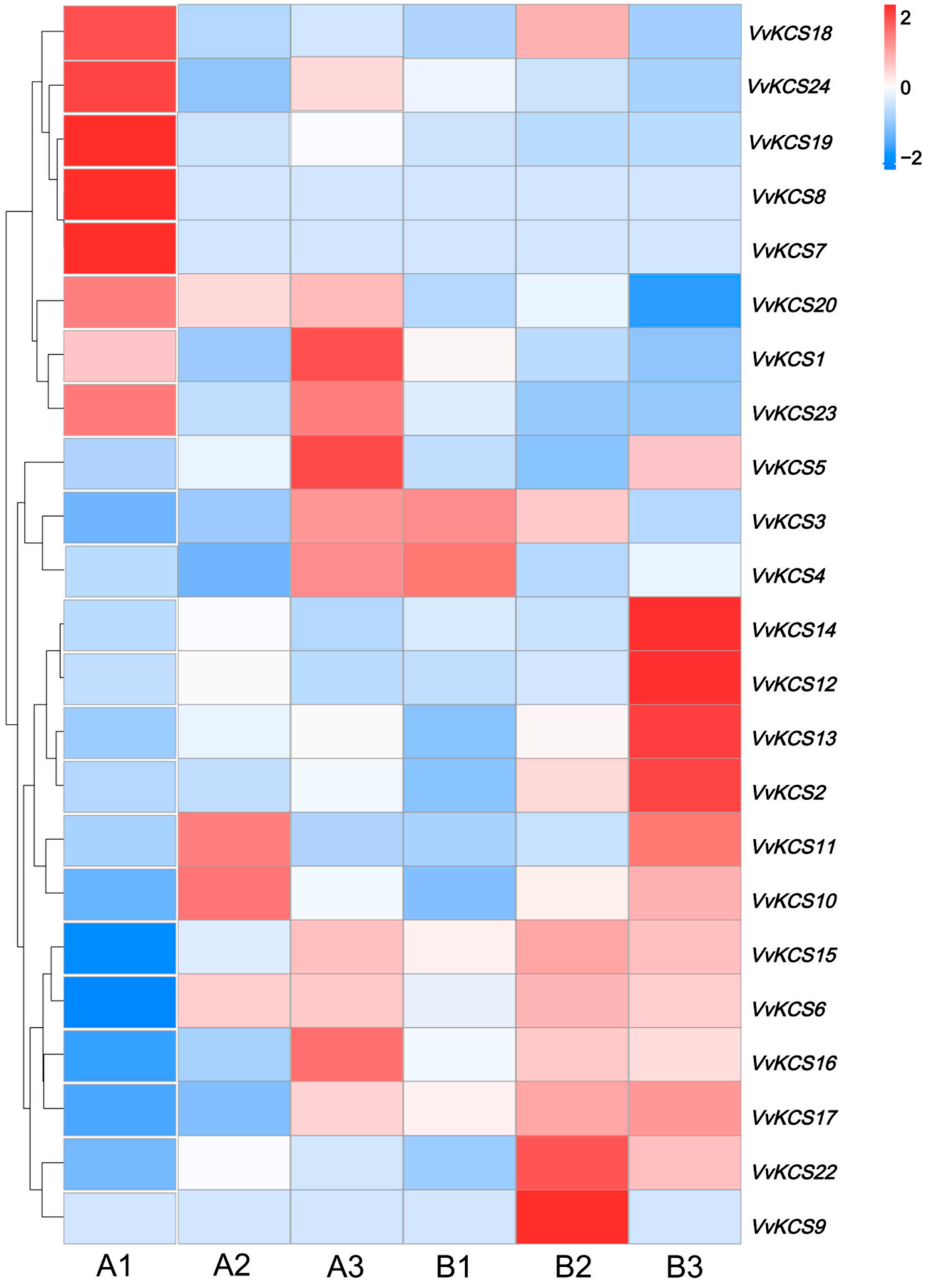

Figure S2. Expression heatmap of *VvKCS* genes in grapevine. A1, 'Y-14' of meiosis stage. A2, 'Y-14' of the tetrad stage. A3, 'Y-14' of the early mononuclear stage. B1, 'Shine Muscat' of the meiosis stage. B2, 'Shine Muscat' of the tetrad stage. B3, 'Shine Muscat' of the early mononuclear stage.

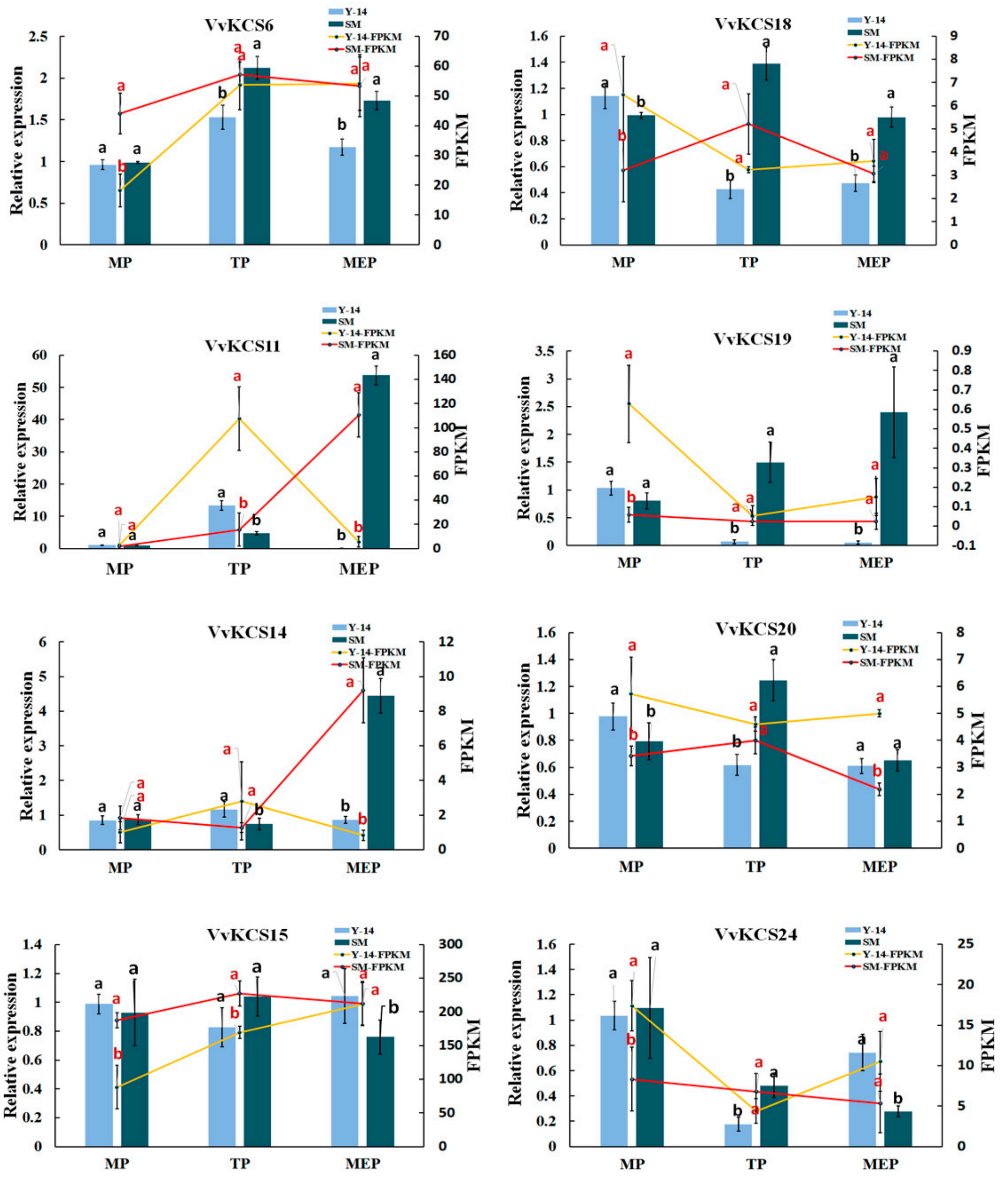

Figure S3. RT-qPCR was used to confirm the relative expression levels of *VvKCS* genes in RNA-seq data. The left y-axis shows the relative gene expression levels determined by RT-qPCR, while the right y-axis shows the FPKM levels determined by RNA-seq. Lower-case letters represent a significant difference (P≤0.05) between 'Y-14' and 'SM' (Shine Muscat), as demonstrated by Student's t-test. Black lower-case letters

represent significant differences between RT-qPCR results and red lower-case letters represent significant differences between FPKM results.

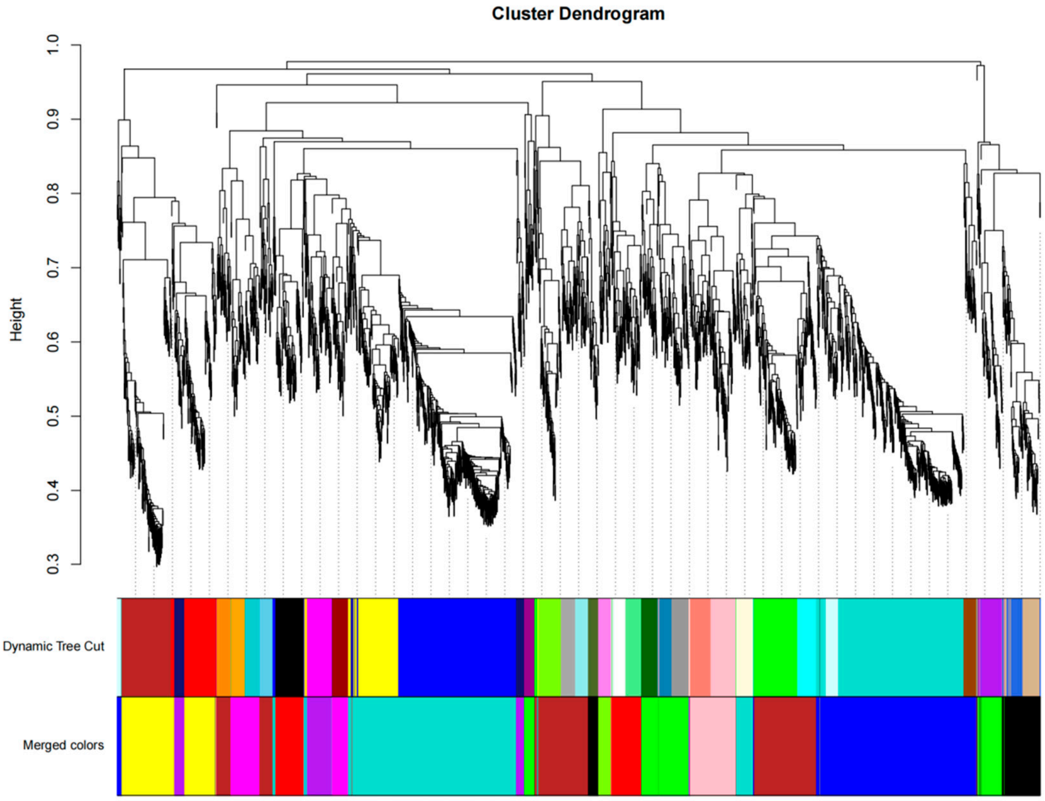

Figure S4. Dendrogram of network analysis modules identified by weighted gene co-expression network analysis (WGCNA).
